# Regimes of Flow over Complex Structures of Endothelial Glycocalyx: A Molecular Dynamics Simulation Study

**DOI:** 10.1038/s41598-018-24041-7

**Published:** 2018-04-10

**Authors:** Xi Zhuo Jiang, Muye Feng, Yiannis Ventikos, Kai H. Luo

**Affiliations:** Department of Mechanical Engineering, University College London, Torrington Place, London, WC1E 7JE UK

## Abstract

Flow patterns on surfaces grafted with complex structures play a pivotal role in many engineering and biomedical applications. In this research, large-scale molecular dynamics (MD) simulations are conducted to study the flow over complex surface structures of an endothelial glycocalyx layer. A detailed structure of glycocalyx has been adopted and the flow/glycocalyx system comprises about 5,800,000 atoms. Four cases involving varying external forces and modified glycocalyx configurations are constructed to reveal intricate fluid behaviour. Flow profiles including temporal evolutions and spatial distributions of velocity are illustrated. Moreover, streamline length and vorticity distributions under the four scenarios are compared and discussed to elucidate the effects of external forces and glycocalyx configurations on flow patterns. Results show that sugar chain configurations affect streamline length distributions but their impact on vorticity distributions is statistically insignificant, whilst the influence of the external forces on both streamline length and vorticity distributions are trivial. Finally, a regime diagram for flow over complex surface structures is proposed to categorise flow patterns.

## Introduction

Flow patterns on surfaces grafted with complex (say brush-like) structures have attracted much attention in the past decades due to their pivotal roles in both engineering^1^, like fabrication of microfluidic and nanofluidic devices^2–4^, and biomedical applications^5,6^ (e.g. drug delivery^7^ and organ culture^8^). A commonly studied example of a brush-like soft matter surface is a polymer brush surface - a surface composed of polymer chains end-grafted onto a solid substrate, and the polymer chains can be stretched away from the surface when high-speed flow passes by^9^.

Theoretical and experimental studies on interfacial flow over the past decades have mainly focused on smooth surfaces. However, smooth surfaces are rarely seen in the physical world. Therefore, it is of great significance to unveil the fluid dynamics in the presence of complex obstacles on coarse surfaces. The past ten years witnessed the germination of flow dynamics over real surfaces. Lanotte *et al*. experimentally investigated the velocity profile of water in a microchannel coated with a grafted hydrophilic polymer brush and observed a slowdown of the flow with the swelling of the polymer brush compared to that of a bare channel^10^. They explained that the drag increase may result from the decrease of the channel width after grafting as well as the brush polydispersity and to flow perturbation generated by the shear of the grafted chains. Charrault *et al*. investigated the boundary conditions for flow of a Newtonian liquid over soft interfaces by measuring hydrodynamic drainage forces with colloid probe atomic force microscopy in a viscous liquid, and proposed mechanism for slip on polymer brush surfaces^11^. Retrospect of fundamentals and applications of polymer brush-modified membranes with an emphasis on tuning the membrane performance through polymer brush grafting under flow shear stress can be found in the review paper by Keating *et al*.^12^. These studies do contribute to our understanding about hydrodynamics over real surfaces. However, compared with their smooth-surface counterpart, the studies are too sparse, and immense studies are still required to pursue the ‘holy grail’ of the fluidics in real physical world.

The biological analogue for synthetic polymer brushes is the endothelial glycocalyx layer (EGL)^13^. The EGL, macromolecular carbohydrate extracellular matrix made up of proteoglycans and glycoproteins, is a functional layer coating on the surface of the entire vascular endothelial cells^14,15^, like in lungs^16,17^. The EGL is the first barrier in direct contact with blood^18^, and acts as a modulator for permeability in the capillary water exchange^19^, as a mechanotransducer of fluid shear stress^20,21^, and as a blood cell interaction regulator^22^. The understanding of flow inside the EGL will shed light on pathologies of many cardiovascular and renal diseases or illness, such as diabetes^23^, ischemia/reperfusion^24^, and atherosclerosis^25^.

As the computational power increases^26^, molecular dynamics (MD) simulations are usually adopted to unveil the atomic events at nanoscales due to the feasibility to trace the trajectories of all atoms by solving Newton’s Second Law of Motion. In this research, large-scale MD simulations will be conducted to study the flow profile over the complex surface structures of the EGL. Four cases involving varying external forces and modified glycocalyx configurations will be constructed to reveal fluid behaviour under intricate circumstances. Finally, flow regimes over complex surface structures will be discussed, hopefully, to provide new insights into flow pattern category in the nanoscale fluidics.

## System Construction and Case Set-up

**Figure 1:**
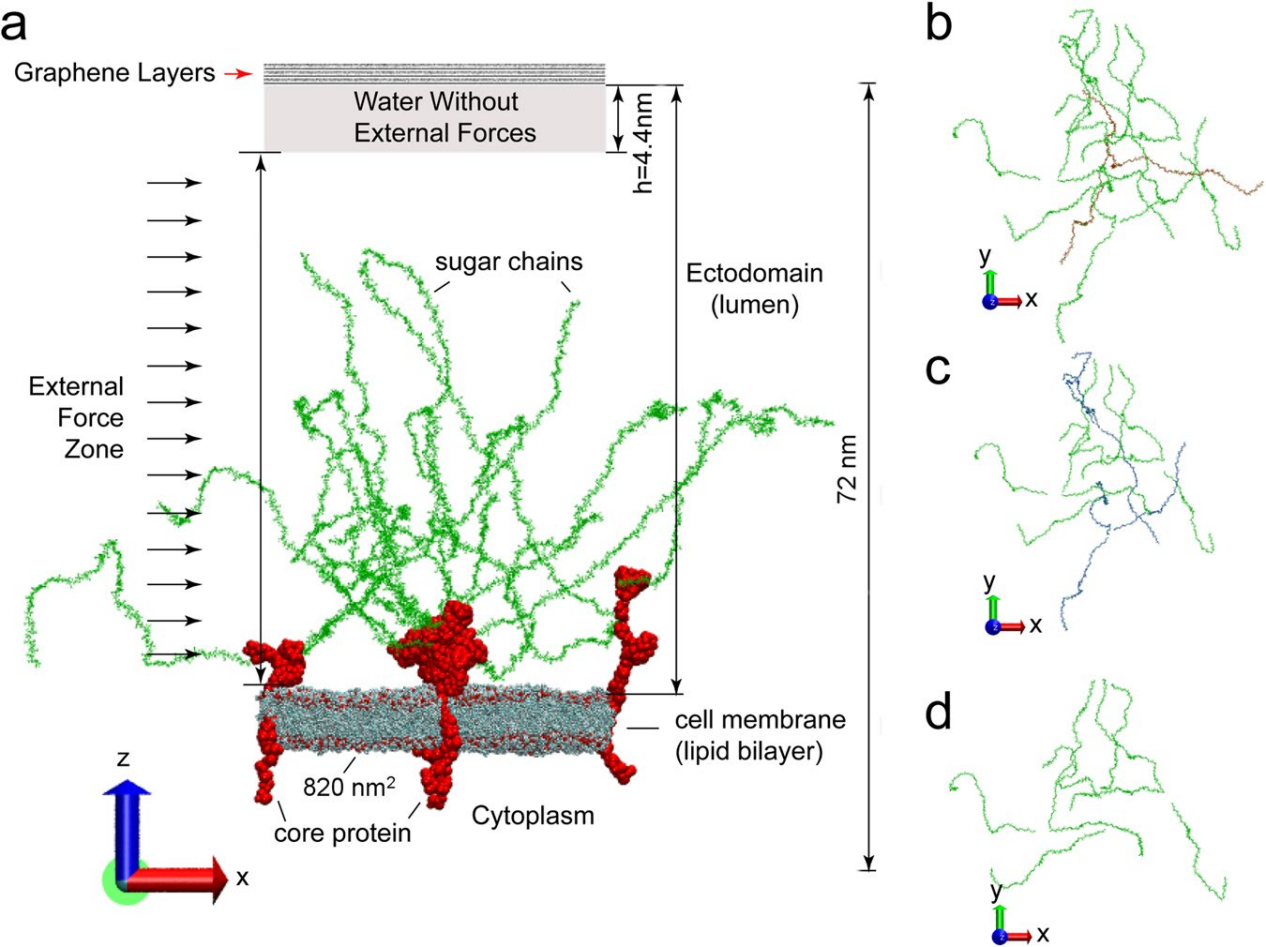
Configurations of the all-atom flow/glycocalyx systems involved in this research. (**a**) Front view for the complete configuration of the flow/glycocalyx system. The system includes 3 Syn-4 dimers as proteoglycan, 18 H S sugar chains attached on the apexes of Syn-4 dimers and a lipid bilayer. External forces are imposed in the *x* direction on the water molecules in the ectodomain. Water molecules and ions are not shown. Graphene layers are added on the top to prevent the water molecules propagating from ectodomain to the cytoplasm (and vice versa) due to the periodic boundary conditions. (**b**) Top view of sugar chain layout in the complete flow/glycocalyx system for Cases A and B in Table 1. (**c**) Top view of a reduced flow/glycocalyx system (Case C in Table 1) with three sugar chains (highlighted red in Panel b) removed from the central glycocalyx element. (**d**) Top view of a reduced flow/glycocalyx system (Case D in Table 1) with half of sugar chains removed (highlighted sugar chains in Panels b and c). In Panels b, c and d, only sugar chain layouts are illustrated.

Flows on a patch of the EGL are studied in this research. The initial configuration of the flow/glycocalyx system is illustrated in Fig. 1a. In the system, Syndecan-4 (Syn-4) proteoglycan and heparin sulfate (HS) sugar chains are selected to model the glycocalyx with the current most detailed structure information^27^. The glycocalyx structure can be divided into three parts: Syn-4 ectodomain linked with the HS sugar chains; Syn-4 transmembrane dimer embedded into a lipid bilayer; and Syn-4 cytoplasmic dimer.

The overall space can be broken down into two compartments by the lipid bilayer of the endothelial cell membrane. Above the lipid bilayer is the ectodomain, representing the lumen where flow passes by. This region contains HS sugar chains, Syn-4 ectodomain in connection with HS sugar chains, water molecules and ions. Below the lipid bilayer is the cytoplasm, representing the inner space of the cell, which is filled with Syn-4 cytoplasmic protein, water molecules and ions. All the biomolecules are solvated and ionized to 0.1 M NaCl aqueous solution. In our simulations, periodic boundary conditions are applied to all the three directions. The *z*-direction periodic motion of water molecules would result in recycling of molecules between the ectodomain and the cytoplasm via upper and lower boundaries. To prevent this from disturbing the micro environments of the ectodomain and cytoplasm, graphene layers are added on the top of ectodomain. This strategy has also been applied in previous studies^27–29^.

To mimic physiological flow, external forces are imposed on the water molecules in the ectodomain. By systematically changing the external forces on water oxygens in the ectodomain, we found that with external forces of 0.002 fN~0.0035 fN, the average bulk flow velocity in the *x* direction, ${\bar{v}}_{x}$, assumes reasonable values of a few cm/s, as discussed in previous studies^28,29^. Thereafter, we selected 0.003 fN as an external force (f = 0.003 fN) to generate flow.

To study flow regimes under various scenarios, four cases are established as listed in Table 1. In Case A, the flow/glycocalyx system contains three glycocalyx elements. Each glycocalyx element comprises three proteoglycans which individually consist of one Syn-4 dimer embedded in the lipid bilayer. Additionally, each proteoglycan has six sugar chains attached to the dimer apexes, so the overall system contains 18 sugar chains. For further analysis, Case B with the same initial configuration as that of Case A is established. In this case, external forces are only imposed for the starting 6 ns to generate the initial velocity for the flow. Thereafter, external forces are removed, leaving the free development of the flow. Throughout the entire simulation of Case B, the glycocalyx configuration remains intact. To study flow patterns under various obstacle layouts, the configuration of the dendritic glycocalyx has been modified by varying the number of sugar chains. After removing 6 and 3 sugar chains, Cases C and D which retain 15 and 9 sugar chains, respectively, are constructed, and the external force in both cases is 0.003 fN. The sugar chain layouts (top views) of Cases A(B), C and D are illustrated in Fig. 1b to d.

**Table 1:** Cases in this research with technic details.

Case	Simulation Time/ns	External Force	No. of Sugar Chains
A	30	0.003 fN (entire simulation)	18
B	24	0.003 fN (for starting 6 ns only)	18
C	15	0.003 fN (entire simulation)	15
D	15	0.003 fN (entire simulation)	9

The simulation box of each case is a hexagonal prism with an area of 820 nm^2^ and height of 72 nm, as described in our previous publication^29^. The four flow/glycocalyx cases individually comprise about 5,800,000 atoms.

## Results

### Temporal Velocity Evolutions

**Figure 2:**
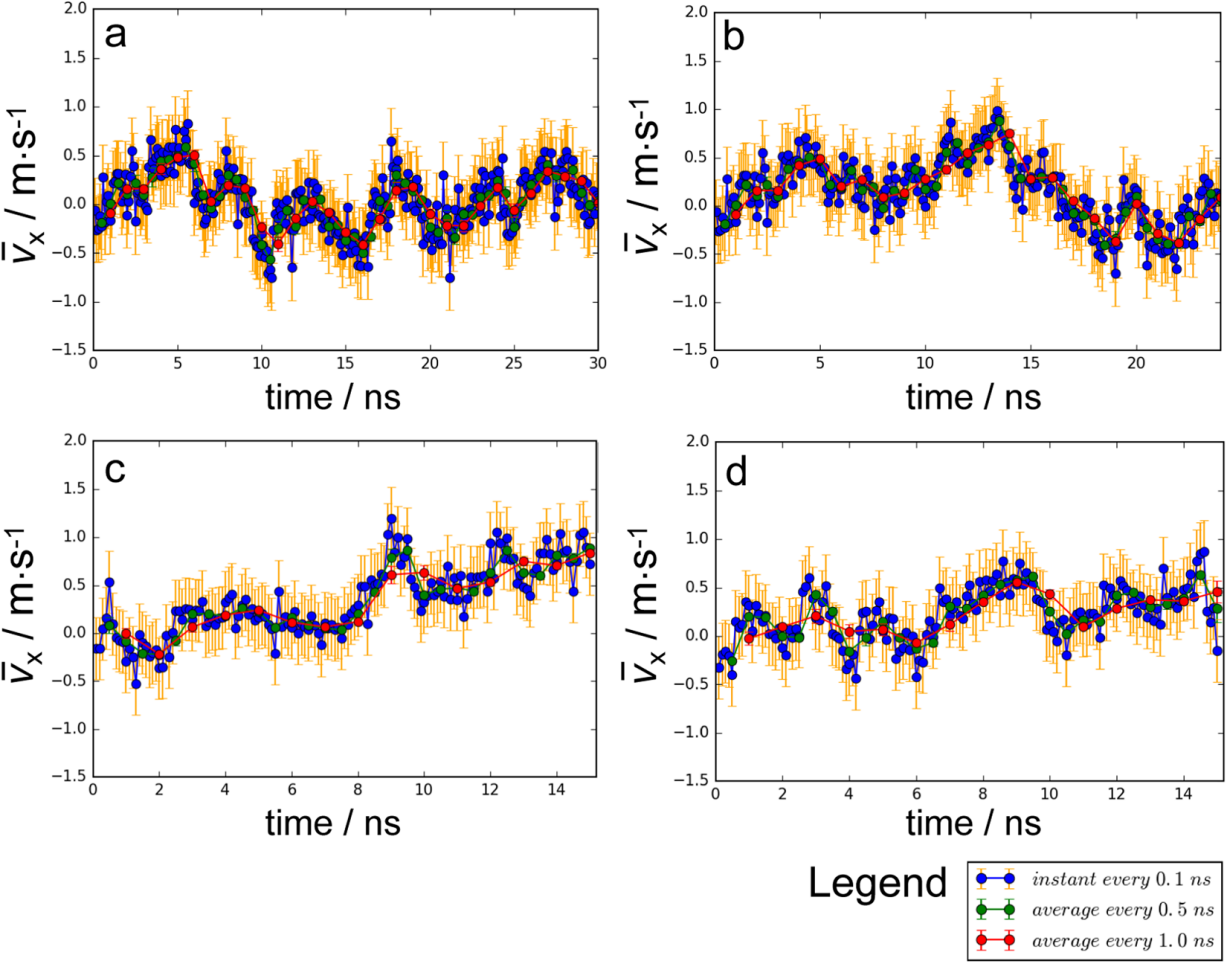
Time-evolutions of bulk flow velocities in the four cases proposed in this research. Panels a to d are time evolutions of velocities for Cases A to D, respectively.

By averaging the *x*-direction velocities of all the ectodomain water molecules in each time-step, instant bulk flow velocity in the *x* direction can be obtained. Instant bulk flow velocity is calculated every 0.1 ns, and time averaging of the instant velocities is also conducted every 0.5 ns and 1.0 ns. Velocity evolutions with time of the four cases in Table 1 are illustrated in Fig. 2.

In Case A (shown in Fig. 2a), the bulk flow velocity fluctuates in the vicinity of 0, which indicates a balance between the imposed external force and internal force stemming from the interactions between the water molecules and glycocalyx biomolecules. While, in Case B (shown in Fig. 2b), after the 6-ns exertion of external forces on ectodomain water molecules, the ectodomain bulk flow experiences an increase in velocity due to inertia until 14 ns, followed by velocity diminution to 19 ns. Thereafter, the velocity trends to equilibrium around 0. In Cases C and D (as shown in Fig. 2c and d), the slightly increasing velocity trends can be attributed to the reduced sterically hindrance from the diminishing sugar chains.

### Spatial Velocity Distributions

**Figure 3:**
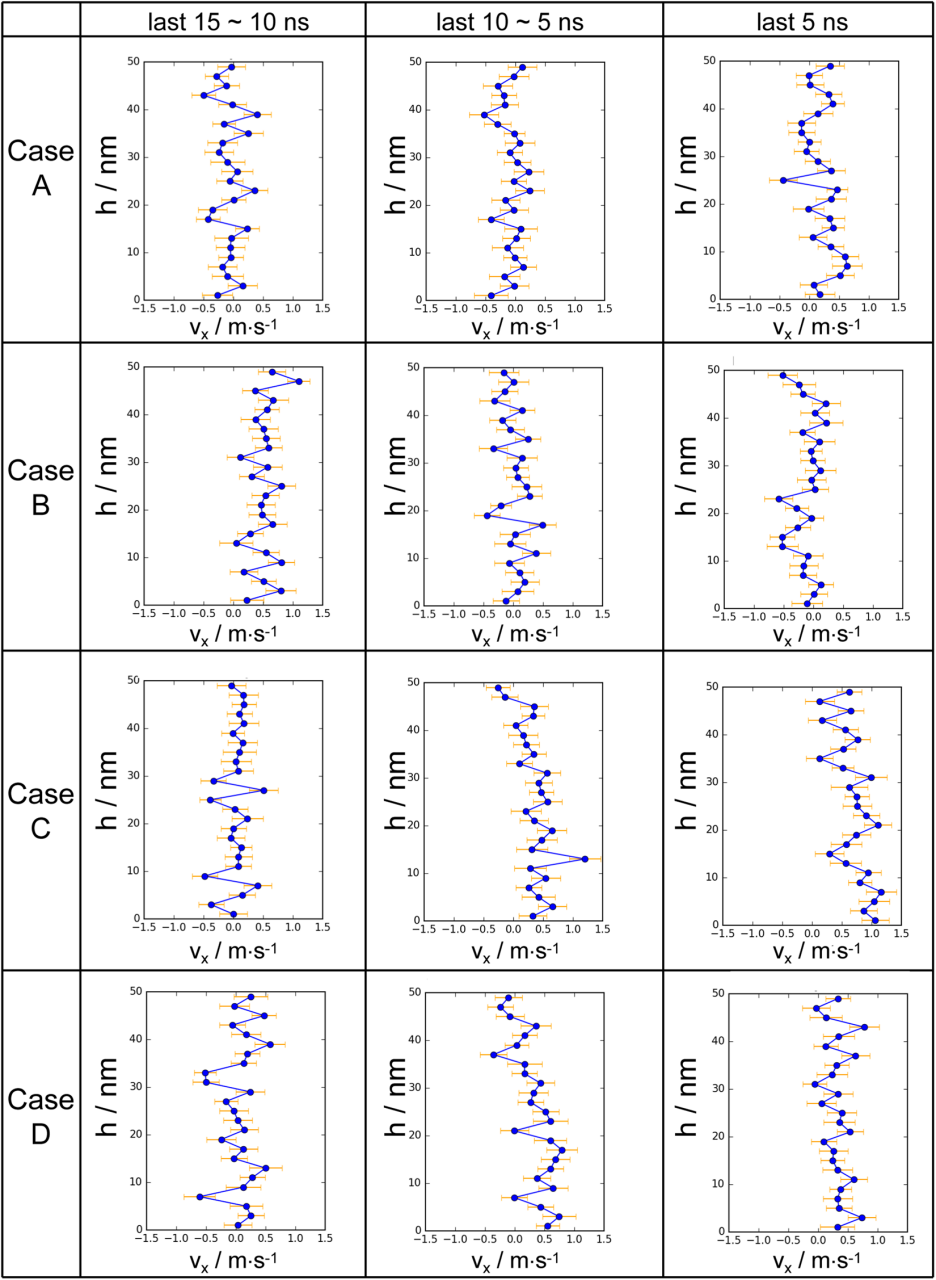
Fluctuating velocity distributions along the height at consecutive instants during the last 15 ns for the four cases proposed in this research.

The spatial velocity distributions, in the ectodomain, of the four cases are also scrutinised. As practised in previous studies^28,29^, a space with 50 nm of height above the height origin of the ectodomain is sliced into 25 equal bins. By averaging the axial velocities of water molecules in each layer every 5 ns, spatial velocity distributions along the height can be obtained as shown in Fig. 3. To facilitate comparisons, last 15-ns results of four cases are adopted in this research.

As illustrated in Fig. 3, the *x*-direction velocity exhibits fluctuating distributions along the height in the ectodomain in all the four cases which may due to the disturbance of the flexible movement of the soft glycocalyx biomolecules. Especially for the last 5 ns, in Case A and B, velocities along the height mostly fluctuate in the vicinity of 0~0.5 m/s and −0.5~0.2 m/s, respectively, whilst in Cases C and D, the velocities fluctuate around greater values (0.5~1 m/s). The differences in the mean of velocities along the height among the four cases are consistent with the velocity evolutions depicted in Fig. 2. The stratified velocities further reveal that the bulk flow velocity variations in Fig. 2 stem from local velocity changes of each layer.

Interestingly, in Cases C and D, backward inclinations (small velocities in the upper region and large velocities in the lower region) of velocity distributions can also been observed. This can be explained by the intricate construction of the system. From the perspective of the *in silico* setup, to prevent the *z*-direction periodic motion of water molecules from disturbing the micro environment of the ectodomain and cytoplasm, graphene layers have been added at the top of the system. Meanwhile, external forces are only exerted on part of the ectodomain region (Fig. 1a), which indicates that water molecules in the remaining region without external forces trend to slow the mainstream by forming a stationary boundary zone. The velocity deviations between the stationary boundary region and the mainstream would cause a backward inclination, and this would aggravate as the mainstream velocity increases. In Cases C and D, the removal of sugar chains accelerates the mainstream in the force-imposed region by reducing the steric hindrance to water molecules, and the backward inclinations are obvious. In fact, slight inclination can also be observed in Cases A and B near the graphene layers of the ectodomain. It is noteworthy that the introduction of graphene layers has not affected the flow patterns on complex surfaces, which are the research focus.

### Streamline Distribution

To examine the influences of the external force and the glycocalyx configurations on 2D flow profiles, streamline distributions of the four cases at the height of 24 ± 1 nm from the ectodomain origin have been extracted. At this height, biomolecules only include flexible sugar chains, and according to the experimental design, the structural differences merely reside in the number of sugar chain molecules among Cases A(B), C and D.

**Figure 4:**
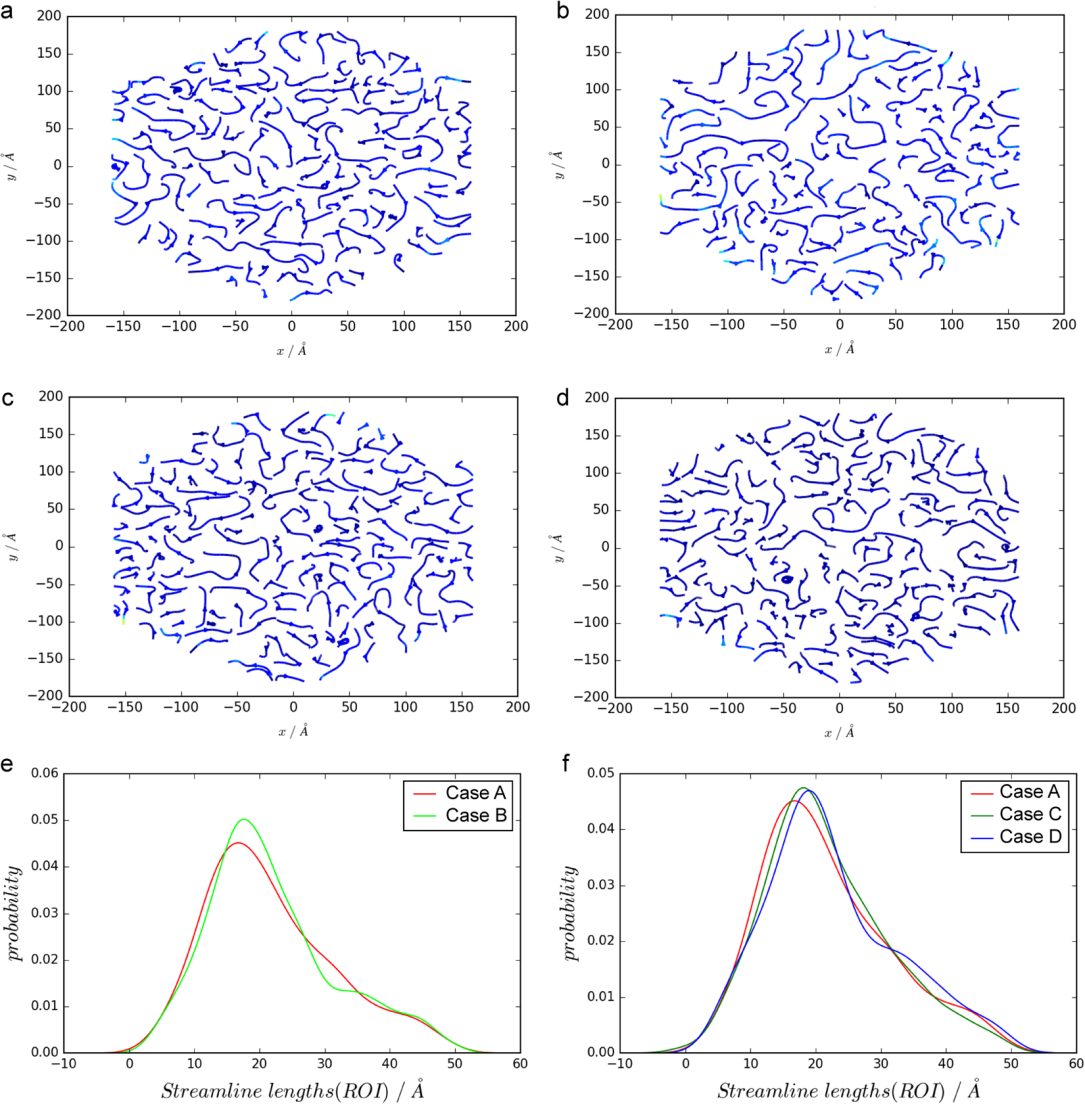
Streamlines at the height of 24 ± 1 nm in the ectodomain and the streamline length probability density distributions. Streamlines are obtained from data of the last 1 ns simulations of the four cases. Panels a to d are streamline distributions for Cases A to D, respectively. Panel e: comparisons of probability density of streamline lengths between Cases A and B. Panel f: comparisons of probability density of streamline lengths among Cases A, C and D.

MD results present discrete streamline distributions in nanoscale fluidics, as shown in Fig. 4a to d. A central region with the *x* coordinate ranging from −88 Å to 88 Å and *y* from −99 Å to 99 Å is the region of our interest (ROI), and the streamline lengths of the ROI of each case have been investigated. Probability density distributions of streamline lengths of ROI are calculated in the four cases. Comparisons between Cases A and B imply that the effect on the streamline lengths from the external force is statistically trivial (with p-value for Cases A and B being 0.35 by a Kolmogorov–Smirnov test), albeit slight discrepancies in the probability density curves can be observed in Fig. 4e. Comparisons of probability density distributions of the streamline lengths in the ROI among Cases A, C and D (Fig. 4f) show that streamline lengths with peak probability density have larger value in the reduced sugar chain cases (Cases C and D) than those in the intact configuration (Case A), which indicates that sugar chains break the streamlines as mass cannot cross streamlines. Kolmogorov–Smirnov (K-S) tests further statistically prove that the probability density difference becomes significant as the number of sugar chains decreases with p-values being 0.44 for Cases A and C, and 0.04 for Cases A and D.

### Vortex Distribution

**Figure 5:**
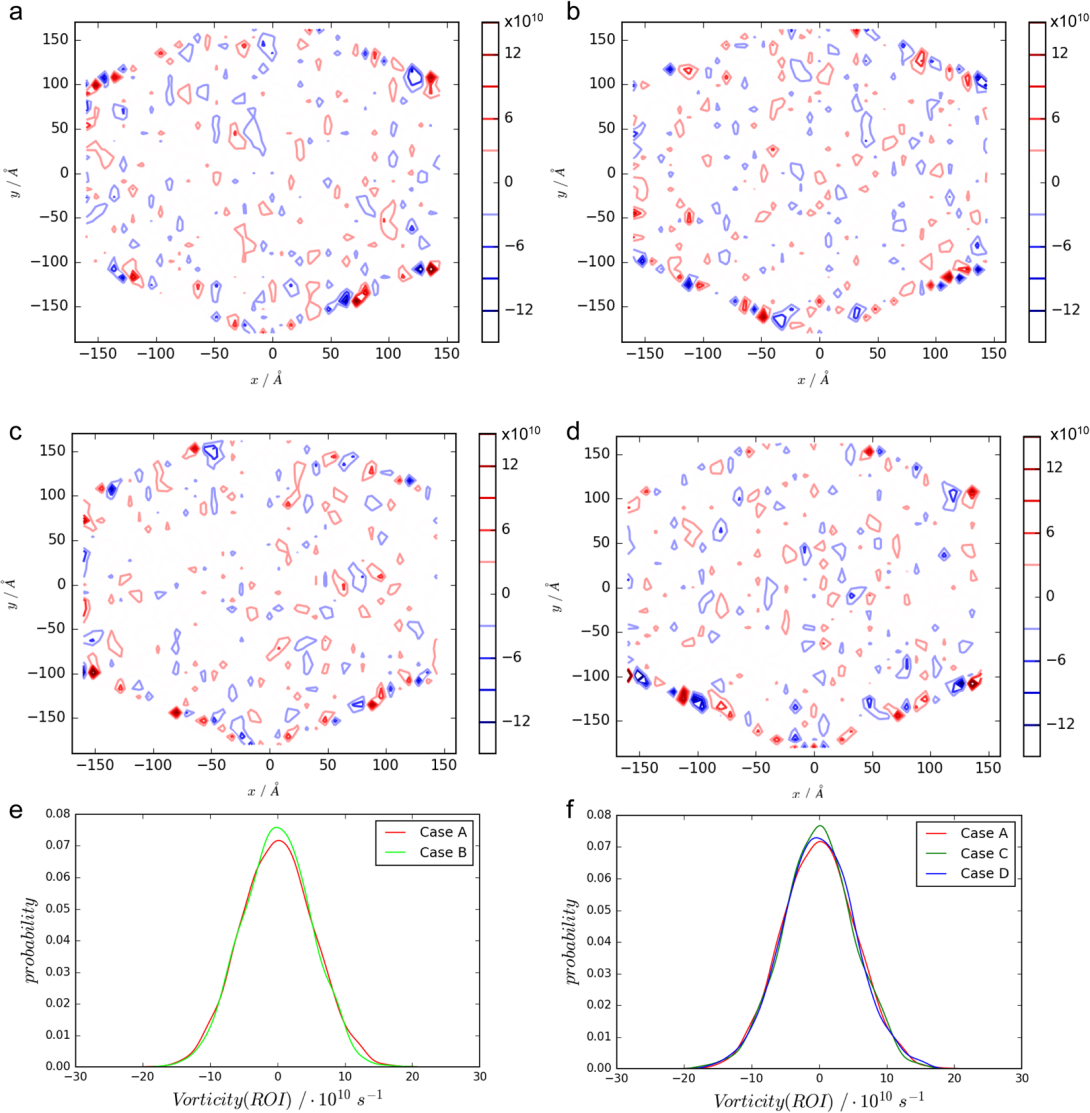
Vortices at the height of 24 ± 1 nm in the ectodomain and vorticity probability density distributions. Vortices are averaged from data of the last 1 ns simulations of the four cases. Panels a to d are vortex distributions for Cases A to D, respectively. Panel e: probability density comparisons between Cases A and B. Panel f: probability density comparisons between Cases A, C and D.

Analogously, vortices at the height of 24 ± 1 nm of the four cases are calculated. Outwards and inwards vortices are interlaced in the four situations (Fig. 5a to d). However, insignificant discrepancies can be found in the probability density distributions of vorticity (strength of vortex) in the ROI between Cases A and B (with p-value being 0.53 by K-S test), which indicates a statistically trivial influence of the *x*-direction external forces (or accelerating velocities in the *x* direction) on vorticity. Indeed, the independence of the vorticity can be explained from its definition (Eq. (3a) in Methods). External forces can significantly change the absolute values of *v*_*x*_ without tremendous changes in its gradient along the *y* direction, resulting in the insignificant differences in the probability density distributions of vorticity between Cases A and B as illustrated in Fig. 5e.

Meanwhile, close probability density distributions of vorticity are observed among Cases A, C and D (with p-value being 0.55 for Cases A and C and 0.79 for Cases A and D), which also implies that the configuration changes in sugar chains have minuscule influences on vorticity. The insignificant effects can be also interpreted via Eq. (3a): changes in sugar chain configurations modify both the *x* and *y* direction velocities simultaneously, resulting in unpredictable variations of the vorticity. Thus, probability density distributions of vorticity are undistinguishable among the intact and reduced sugar chain configuration cases.

## Discussion - Flow Regimes over Surface with Complex Structures

**Figure 6:**
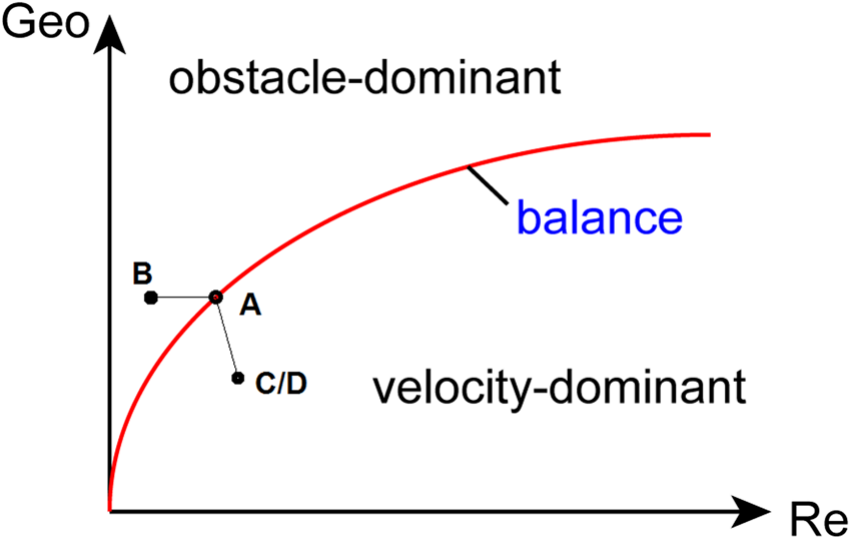
Flow regimes over the surface with complex structures. Cases A to D are labelled in the diagram according to their Geo and Re numbers. Only the number of sugar chains is considered in Geo here for simplification.

The velocity distributions reported in this research and the relation between flow and the glycocalyx configurations discussed in the previous study^29^ suggest that flow patterns on complex surfaces with soft obstacles depend on both the energy carried by the flow per se and the steric hindrance from the obstacle. Figure 6 proposes a diagram describing the flow regimes over the surface with complex structures. In Fig. 6, the Reynolds Number (Re), defined as the ratio of inertial forces to viscous forces within a fluid and usually calculated by Eq. (1), is used as an indicator to describe the energy carried by the flow.

1 $$\mathrm{Re}=\frac{vL}{\upsilon }$$

where *v* is the velocity of the flow, *L* is the characteristic length, and *υ* is the kinematic viscosity. Additional indicator depicting the geometric information (Geo) of the soft obstacles is employed to quantify the steric hindrance from the biomolecular obstacles. The Geo number can be a function of the number of sugar chains in this case as well as the sugar chain layouts, as generally expressed in Eq. (2).

2 $${\rm{Geo}}=f(N,{x}_{1},\,\mathrm{...},\,{x}_{i})={a}_{0}N+\sum _{i}{a}_{i}{x}_{i}$$

In Eq. (2), *N* is the number of sugar chains, *x*_1_ to *x*_*i*_ represent the potential geometric elements of the obstacles and the elements are selected by requirement. The coefficients *a*_0_ to *a*_*i*_ are the weight coefficients of the geometric parameters. A balance curve can be obtained by a set of Re and Geo numbers in situations when the flow energy is counteracted by the steric hindrance. For instance, for Case A in this research, the bulk flow velocity fluctuates around some value, implying a force balance between the external forces and hindrance from the biomolecules. Correspondingly, the Re and Geo numbers can be calculated in this circumstance. The balance curve then divides the flow regime diagram into two regions: the obstacle-dominant region and the velocity-dominant region. As self-explained, in the velocity-dominant region, the energy conveyed by the flow overwhelms the energy dissipation from the steric hindrance, sometimes accompanying conformational changes of the soft obstacles in the direction of the flow^29^. In the obstacle-dominant region, the flow energy is dissipated by the obstacles via their interactions, and the flow will eventually reach a static equilibrium state.

The four cases mentioned in this research can be catalogued into three regimes accordingly, and are labelled in Fig. 6. To facilitate analysis, only the number of the sugar chains is considered in the Geo number (i.e. Geo = *N*) here. As mentioned previously, in Case A, the velocity oscillates around a certain value, representing a balance state on the balance curve. In Case B, the geometric structure of the obstacles remains intact, leaving the Geo number unchanged. Moreover, the release of the external forces leads to velocity diminishing, so Case B is labelled on the left to Case A. As for Case C (or D), the reduction in sugar chain number gives a decreased Geo number. On the other hand, the reduced resistance by the removal of sugar chains facilitates the flow and results in an ascending Re number. Therefore, Case C (or D) is spotted on the lower-right to Case A.

In this research, large-scale molecular dynamics simulations were conducted to study the flow profiles under the complex structures of the endothelial glycocalyx layer. Four cases involving varying external forces and modified sugar chain configurations were constructed to reveal fluid behaviour under intricate circumstances. Temporal evolutions and spatial distributions of the ectodomain flow were scrutinised for the four cases, and the effects of external forces and sugar chain configurations on flow were discussed as described in the Results. Streamlines and vortices were also illustrated, and results showed that sugar chains configurations affect streamline length distributions but their impact on vorticity distributions is statistically insignificant whilst the effects of external forces on both distributions are statistically insignificant. Finally, a flow regime diagram, in terms of the Reynolds number and geometric information, was constructed to categorise flow over complex structures. Furthermore, the four cases constructed in this research were labelled in the diagram. This research contributes to understanding flow regimes over complex surface structures and provides new insights into nanoscale fluidics inside the endothelial glycocalyx layer. In the future work, additional *in silico* experiments are expected to complete the flow regime diagram over the surface with complex structures.

## Methods

### Protocol Details of Molecular Dynamics Simulations

The TIP3P water model^30^ is adopted to simulate water molecules. A CHARMM biomolecular force field^31^ has been applied to the proteins and the lipid bilayer. Force field parameters for sugar chains and graphene layers are adopted from a previous study^27^.

In each case, after building up or revising the biomolecular configurations, a simulation in NPT ensemble with graphene layers being fixed was conducted at 1 atm and 310 K using a Langevin thermostat and a Nosé-Hoover Langevin piston for 2 ns, followed by a simulation in NVT ensemble using a Langevin thermostat to maintain temperature at 310 K for 0.5 ns. The last frame of the NVT simulation was then used as the initial configuration (e.g. as shown in Fig. 1a) of the follow-up “production” flow simulations. In the flow simulations, the Lowe-Andersen thermostat^32^ was selected to maintain the temperature at 310 K.

In the flow simulations, the velocity Verlet integration method^33^ was used to advance the positions and velocities of the atoms in time. A 2-fs timestep, and particle mesh Ewald electrostatics^34^ with a grid density of 1/Å^3^ were used. The SETTLE algorithm^35^ was used to enable the rigid bonds connected to all hydrogen atoms. The van der Waals interactions were calculated using a cutoff of 12 Å with a switching function starting at 10 Å.

All MD simulations were performed using the software NAMD 2.9^36^. The visualisation of the molecular structures was performed by the VMD^37^ package. Post-processing of the MD results was accomplished using self-developed PYTHON (Python Software Foundation, Wilmington, De) scripts. All parallel simulations and non-visualised post-processing were conducted on ARCHER, UK’s national supercomputing service. To obtain a simulation result with physical time of 1 ns, 9,000 compute cores were simultaneously employed for about 2 hours.

### Calculation of Vorticity

Vorticity is calculated according to Eq. (3a).

3a $$\overrightarrow{w}=(\frac{\partial {v}_{y}}{\partial x}-\frac{\partial {v}_{x}}{\partial y})\overrightarrow{z}$$

where $\overrightarrow{w}$ is the vortex vector, *v*_*x*_ and *v*_*y*_ are velocities in the *x* and *y* directions, respectively. *x*, *y* and *z* are coordinates in three directions.

In the post-processing, to calculate the vorticity of the ROI. The ROI was meshed into 484 grids, in the XoY plane, with the grid dimension of 8 × 9 Å^2^. The velocities in the *x* and *y* directions of each grid were then averaged. Therefore, Eq. (3a) is discretized as shown in Eq. (3b).

3b $${\overrightarrow{w}}_{x,y}=(\frac{{v}_{y,x+{\rm{\Delta }}x}-{v}_{y,x}}{{\rm{\Delta }}x}-\frac{{v}_{x,y+{\rm{\Delta }}y}-{v}_{x,y}}{{\rm{\Delta }}y})\overrightarrow{z}$$

In Eq. (3b), Δ*x* and Δ*y* are the side lengths of each grid with the value of 8 Å and 9 Å, respectively.
